# Carbon-Ion radiotherapy alone for inoperable locally advanced Non-Small cell lung cancer: A Japanese National registry study (J-CROS-LUNG)

**DOI:** 10.1007/s11604-025-01925-z

**Published:** 2026-01-13

**Authors:** Shuri Aoki, Hiroaki Suefuji, Mio Nakajima, Nobuteru Kubo, Osamu Suzuki, Miyako Satouchi, Kimihiro Shimizu, Takehiko Fujisawa, Kensuke Umehara, Hitoshi Ishikawa, Yoshiyuki Shioyama

**Affiliations:** 1 QST Hospital, National Institute for Quantum Science and Technology, Chiba, Japan; 2 Department of Radiology, Cancer Institute Hospital, Japanese Foundation for Cancer Research, Tokyo, Japan; 3 Ion Beam Therapy Center, SAGA-HIMAT Foundation, Saga, Japan; 4 Gunma University Heavy Ion Medical Center, Gunma, Japan; 5 Osaka Heavy Ion Therapy Center, Osaka, Japan; 6 Department of Thoracic Oncology, Hyogo Cancer Center, Hyogo, Japan; 7 Division of General Thoracic Surgery, Department of Surgery, Shinshu University School of Medicine, Nagano, Japan; 8 Department of Health Research, Chiba Foundation for Health Promotion and Disease Prevention, Chiba, Japan

**Keywords:** Carbon-ion radiotherapy, Locally advanced, NSCLC, Radiotherapy alone, Inoperable, Real-world data.

## Abstract

**Purpose:**

Carbon-ion radiotherapy (CIRT) offers high-dose concentration and enhanced biological effects. Since 2016, a nationwide prospective registry study of CIRT for locally advanced non-small cell lung cancer (LA-NSCLC) has been conducted in Japan. This study aimed to evaluate clinical outcomes of CIRT in patients with LA-NSCLC who were ineligible for surgery or chemoradiotherapy (CCRT).

**Materials and methods:**

Patients with inoperable LA-NSCLC treated with CIRT in Japan from May 2016 to June 2020 were included. Most patients received 64–72 Gy in 16 fractions per the Japanese Society for Radiation Oncology (JASTRO) unified policy. Elective nodal irradiation was allowed for nodal disease. No systemic therapy was administered before or after CIRT. Overall and progression-free survival were estimated by the Kaplan–Meier method; local failure was evaluated using the cumulative incidence function (CIF) with Gray’s test. Two-sided *P* < 0.05 was considered significant.

**Results:**

Median follow-up was 28 months for all patients and 36 months for survivors. Of the 55 patients, clinical stages (UICC 8th) were: IIB (26), IIIA (17), and IIIB (12). A clinical diagnosis without histological confirmation was established in four patients (7.3%), and interstitial pneumonia (IP) was present in nine (16.4%). The 3-year overall survival and progression-free survival were 49.1% (95% confidence interval [CI], 33.8–62.7%) and 28.3% (95% CI, 16.6–41.3%), respectively. Using competing risks, the 3-year CIF was 37.4% (95% CI, 24.2–50.5%). No grade ≥ 4 toxicity was observed; grade 2 and 3 pneumonitis occurred in 3 (5.5%) and 2 (3.6%) patients, respectively. In multivariable analysis, concomitant IP was a significant factor for overall survival (*P* = 0.011).

**Conclusion:**

CIRT demonstrated favorable tumor control with acceptable toxicity in patients with LA-NSCLC ineligible for surgery or CCRT. It may be a promising treatment option for this patient population.

**Supplementary Information:**

The online version contains supplementary material available at 10.1007/s11604-025-01925-z.

## Introduction

Concurrent chemoradiotherapy (CCRT) remains the standard treatment for unresectable locally advanced non-small-cell lung cancer (LA-NSCLC). For patients without disease progression or symptomatic radiation pneumonitis following CCRT, maintenance immune checkpoint inhibitors (ICIs) or molecularly targeted drugs have been recommended [1, 2]. Although prognosis has improved with the development of novel systemic therapies, many patients are unable to benefit from these treatments owing to advanced age, poor performance status, or pre-existing comorbidities. In aging populations, the proportion of patients considered frail is expected to increase. For these patients, tumor control using radiotherapy remains essential [3].

Particle therapy is anticipated to play a significant role in the management of such patients. This modality, primarily utilizing carbon-ion and proton beams, offers superior dose concentration, enabling higher doses to the target volume while minimizing risk to surrounding vital organs, such as the lungs, esophagus, trachea, and heart [4, 5]. Carbon-ion radiotherapy (CIRT) provides a steeper dose distribution and greater biological effectiveness, achieving robust local control even in patients ineligible for chemotherapy [6]. The unique physical characteristics of CIRT (such as the Bragg peak and minimal exit dose) enable dose optimization while reducing the number of beam angles, typically employing anteroposterior beams. This approach is hypothesized to reduce the risk of radiation pneumonitis by limiting low-dose irradiation to the lung, which is a concern with multi-field or rotational irradiation techniques.

CIRT was first clinically developed in Japan and has been covered by health insurance in Japan since 2024 as a curative treatment for early-stage NSCLC. However, for patients with LA-NSCLC, CIRT is mainly used as a monotherapy for those who are ineligible for standard CCRT owing to comorbidities. The efficacy and safety of CIRT for LA-NSCLC remain inadequately established, with existing evidence largely derived from single-center studies in Japan. To address this gap, a prospective nationwide registry study was initiated in May 2016. Based on data from this registry, the Particle Radiation Therapy Committee of the Japanese Society for Radiation Oncology (JASTRO) conducted a comprehensive analysis of CIRT in patients with NSCLC. Findings for stage I NSCLC have been previously reported [7, 8]. The present study reports the outcomes of CIRT monotherapy in patients with unresectable LA-NSCLC and evaluates its efficacy and safety using data from this multicenter registry.

## Materials and methods

### Patients

This multicenter prospective registry study utilized data from the Japan Carbon-Ion Radiation Oncology Study Group (J-CROS), which includes all facilities in Japan that provide CIRT. All seven CIRT centers in Japan participate in the J-CROS. Among these, the SAGA-HIMAT Foundation (HIMAT, Saga Prefecture), National Institutes for Quantum Science and Technology (QST, Chiba Prefecture), Gunma University Heavy Ion Medical Center (GHMC, Gunma Prefecture), and Osaka Heavy Ion Therapy Center (HIMAK, Osaka Prefecture) have registered cases for this study.

Patients with LA-NSCLC (TanyN1-3M0 or T3-4N0M0) who were ineligible for surgery or standard CCRT and treated with CIRT monotherapy between May 2016 and June 2020 were included. Clinical stage was determined using the Union for International Cancer Control (UICC) TNM classification, 8th edition [9]. Pathological or cytological diagnosis of lung cancer was obtained whenever possible. Nodal staging was primarily based on imaging, with CT scans being mandatory. FDG-PET was also routinely utilized in almost all cases. Pathological confirmation of nodal disease was not a mandatory criterion. Eligibility for CIRT was determined by a multidisciplinary cancer board at each participating institution. Patients were considered suitable for CIRT monotherapy if they were deemed ineligible for surgery or CCRT due to factors such as advanced age, comorbidities, or tumor characteristics. Specific reasons for ineligibility were not uniformly recorded in the registry. Written informed consent was obtained from all patients prior to participation. This study was registered in the UMIN Clinical Trials Registry (UMIN000024709). The study was conducted in accordance with the principles of the Declaration of Helsinki.

### Treatment

The CIRT dose/fractionation defined by J-CROS and approved by JASTRO [10] was used. Nevertheless, in order to reflect a real-world clinical setting, all patients with LA-NSCLC who received CIRT were enrolled in the analysis, regardless of the dose fractionation schedule. In most cases, patients received a total CIRT dose of 64–72 Gy in 16 fractions. Two patients with T4N0M0 received 60–64 Gy in 4 fractions. The CIRT dose represents the biological effectiveness-weighted dose, calculated using a modified microdosimetric kinetic model [11, 12]. Treatment plans were developed according to institutional protocols, and parameters for target volume, setup margins, and dose constraints for organs at risk were determined accordingly.

Primary lung lesions and metastatic lymph nodes were defined as the gross tumor volume (GTV) on computed tomography (CT) images. In N0 cases, elective nodal irradiation (ENI) was omitted. In N1–3 cases where ENI was indicated, the ipsilateral hilar and/or mediastinal lymph nodes were designated as the extended clinical tumor volume (CTV), i.e. CTV1, and internal margins and setup margins were added to account for respiratory motion, resulting in an expanded planning target volume (PTV), i.e. PTV1. During the replanning period, which varies depending on the facility and case, a reduced CTV with a margin of 5–10 mm around the GTV (i.e. CTV2) and a reduced PTV (PTV2) with a similar margin to PTV1 were created for boost irradiation. CTV margin can be reduced depending on the distance from the organs at risks (OARs). CIRT was delivered using two to five fields in a near-contralateral configuration.

No patients received chemotherapy or ICI in combination with CIRT.

### Treatment evaluation and statistical analysis

In this study, local recurrence was defined as recurrence of the primary tumor within the irradiated field; regional recurrence was defined as nodal failure regardless of field coverage. Diagnoses of local or metastatic recurrence were primarily based on chest radiography and CT, with PET-CT and MRI used as auxiliary tools. Local failure was defined as recurrence of the primary tumor within the irradiated field (i.e., local recurrence). The diagnostic criteria for recurrences were not standardized across institutions and were based on the clinical judgment of each institution.

Overall survival (OS) was defined as the period from the initiation of CIRT to the date of the final follow-up or death. Progression-free survival (PFS) was defined as the period from CIRT initiation to disease relapse or death from any cause. Local control (LC) was defined as the period from CIRT initiation to local relapse.

OS and PFS were estimated using the Kaplan–Meier method, with differences between curves assessed by the log-rank test. For local failure, the cumulative incidence function (CIF) was estimated, treating death without prior local failure as a competing event; group comparisons used Gray’s test. As death is a competing event, CIF was prespecified as the primary measure of local disease control. Kaplan–Meier estimates of LC (treating death as censoring) are provided in the Supplementary Materials for the purpose of historical comparison with retrospective reports. Potential prognostic factors for OS were evaluated using Cox proportional hazards models. Univariable screening used a liberal entry threshold (two-sided *P* < 0.20) to identify candidate variables for the multivariable model, whereas statistical significance for all inferential tests was defined as two-sided *P* < 0.05. Toxicity was graded according to the Common Terminology Criteria for Adverse Events (CTCAE) version 5.0 [13] with the highest grade recorded during the observation period.

## Results

### Patient characteristics

Between May 2016 and June 2020, 55 patients with inoperable LA-NSCLC were treated with CIRT. Patient characteristics are presented in Table 1. The median age was 73 years (range, 49–90). Clinical stage (UICC 8th) was distributed as follows: IIB (26 patients, 47%), IIIA (17 patients, 31%), and IIIB (12 patients, 22%). Pathological or cytological diagnosis of lung cancer was obtained in 51 of 55 patients (92.7%); the remaining four patients (7.3%) were diagnosed clinically. Among the 55 patients included in this study, interstitial pneumonitis (IP) was present in 9 patients (16%), and 16 (29%) were considered unsuitable for conventional X-ray radiotherapy due to tumor extent or comorbidities. The CIRT dose fractionation ranged from 60–72 Gy in 4–16 fractions, with 72 Gy in 16 fractions (n = 32) and 64 Gy in 16 fractions (n = 18) accounting for 91% of all treatments. Second primary cancers in other organs were identified in 16 patients (29%).

**Table 1:** Patient characteristics (n = 55)

Characteristics	n (%) or median (range)
Age (y)	
Median (range)	73 (49–90)
Sex	
Male	44 (80.0)
Female	11 (20.0)
ECOG performance status	
0	30 (54.5)
1	22 (40.0)
2	3 (5.5)
Histology	
Adenocarcinoma	21 (38.2)
Squamous cell carcinoma	23 (41.8)
NSCLC, NOS	6 (10.9)
Others	1 (1.8)
Clinical	4 (7.3)
Primary tumor diameter (cm)	
Median (range)	5.1 (1.5–11.5)
Clinical stage	
2B	26 (47.3)
3A	17 (30.9)
3B	12 (21.8)
T-stage	
T1b	5 (9.1)
T1c	4 (7.3)
T2a	7 (12.7)
T2b	6 (10.9)
T3	24 (43.6)
T4	9 (16.4)
N-stage	
N0	19 (34.5)
N1	14 (25.5)
N2	20 (36.4)
N3	2 (3.6)
Location	
Peripheral	42 (76.4)
Central	13 (23.6)
IP	
Yes	9 (16.4)
No	46 (83.6)
Dose prescription	
60–64 Gy in 4 fr	2
64–68 Gy in 16 fr	18
69.6 Gy in 12 fr	2
70.4–72 Gy in 16 fr	33

ECOG, Eastern Cooperative Oncology Group; NSCLC, non-small cell lung cancer; NOS, non-specific classification; IP, interstitial pneumonia; fr, fraction

### Treatment outcomes

**Fig. 1:**
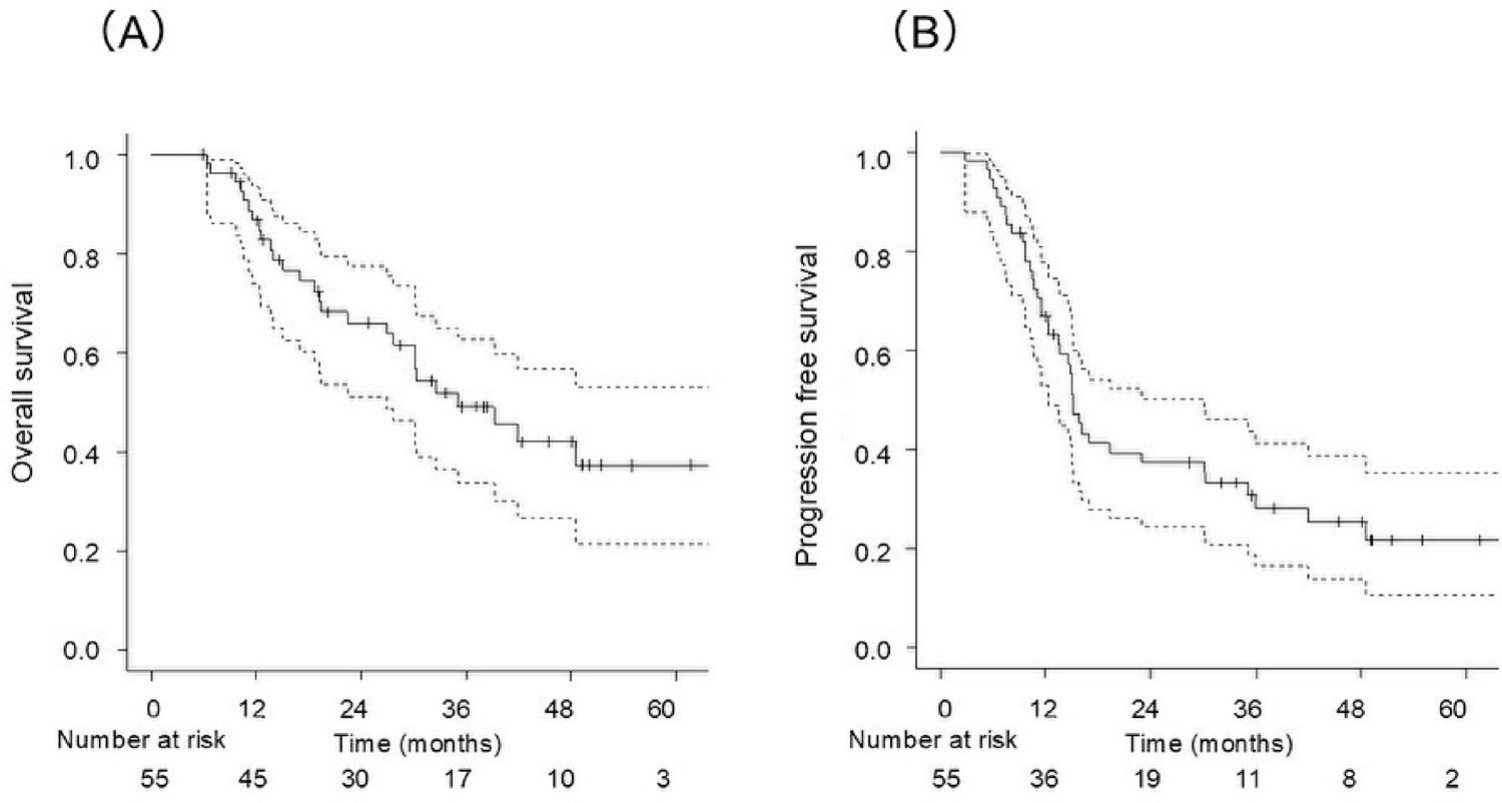
Overall survival (OS, **A**) and progression-free survival (PFS, **B**) estimated by the Kaplan–Meier method in patients treated with carbon-ion radiotherapy (CIRT)

**Fig. 2:**
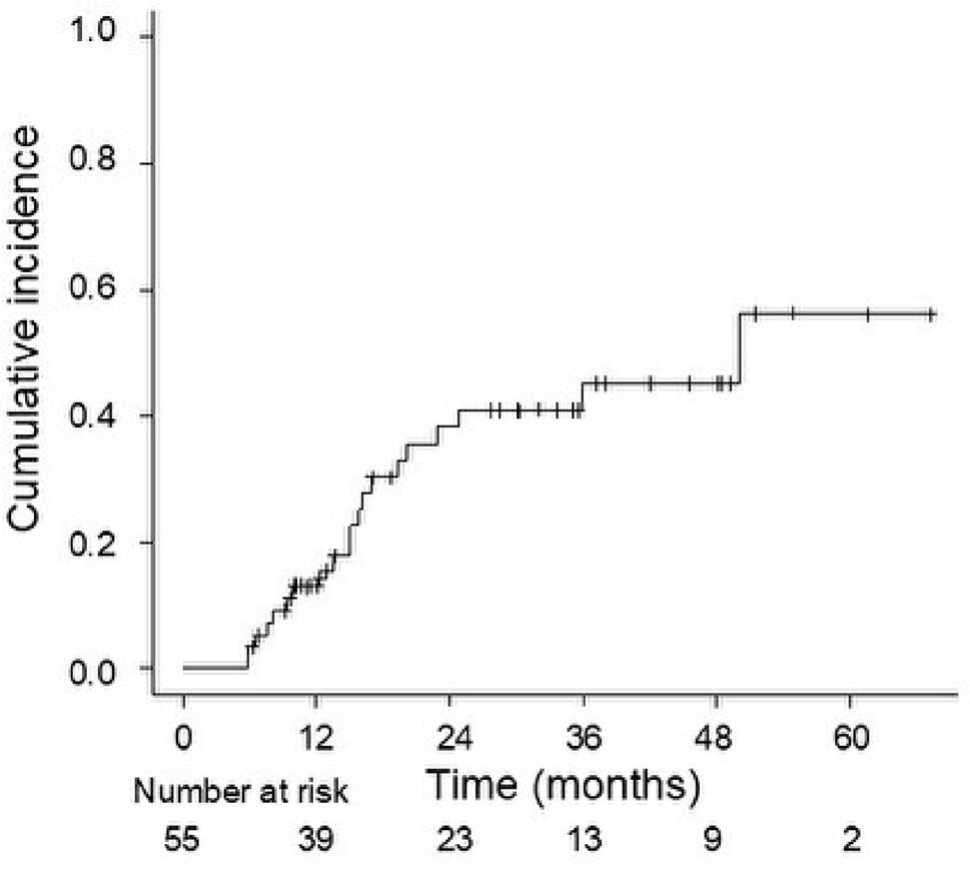
Cumulative incidence function of local failure. The cumulative incidence was estimated using a competing-risks approach, with death without prior local failure treated as a competing event

The median follow-up duration was 27.7 months (5.9–67.1) for all patients and 36.4 months (5.9–67.1) for survivors. The median OS was 35.1 months (range: 5.9–67.1), with 2-year and 3-year OS rates of 66.0% (95% confidence interval [CI], 53.4–79.2%) and 49.1% (95% CI, 33.8–62.7%), respectively (Fig. 1A). Recurrence was observed in 29 patients (53%), with 45 sites of first recurrence: 20 local, 12 regional, and 13 distant. The median PFS was 15.1 months (range, 12.4–30.1), with 2-year and 3-year PFS rates of 37.5% (95% CI, 24.5–50.3%) and 28.3% (95% CI, 16.6–41.3%), respectively (Fig. 1B). Using a competing-risks approach (death without prior local failure as a competing event), CIF at 2 and 3 years was 34.8% (95% CI, 22.2–47.7%) and 37.4% (95% CI, 24.2–50.5%), respectively (Fig. 2). Gray’s test showed no significant difference (*P* = 0.22).

**Fig. 3:**
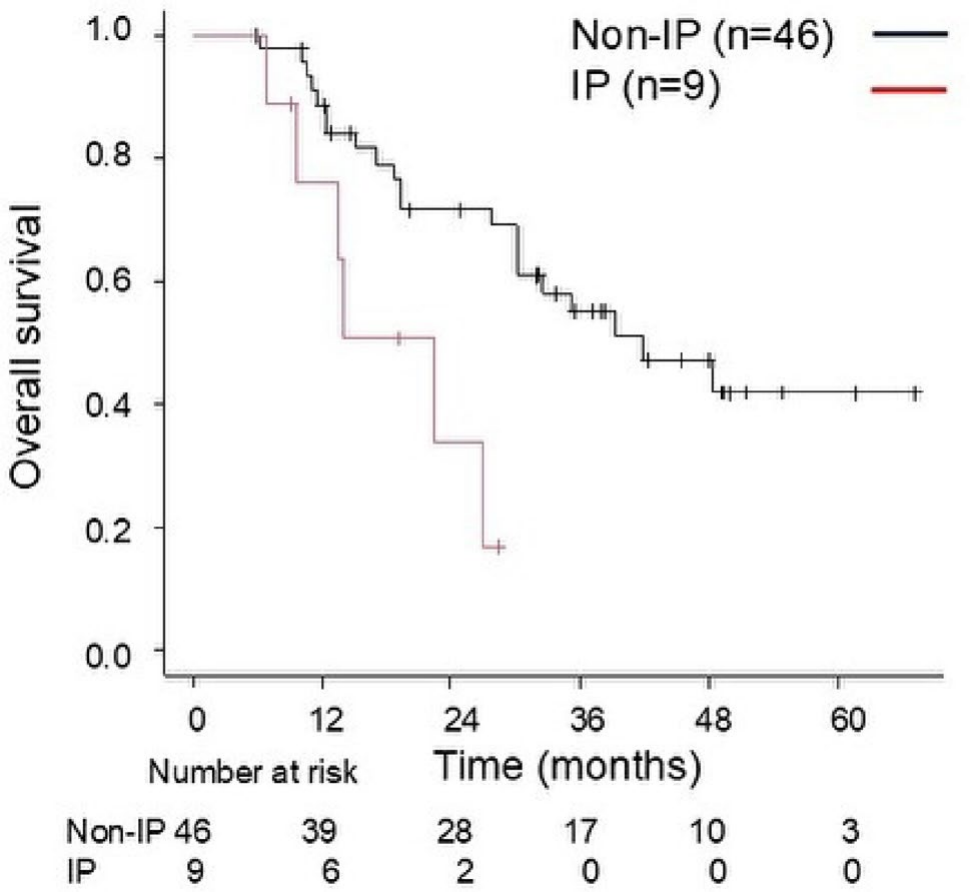
Association between overall survival (OS) and concomitant interstitial pneumonitis

Table 2 summarizes the results of the analysis of OS-related factors. In the univariate analysis, age and performance status were not significant predictors (both *P* ≥ 0.20) and therefore were not entered into the multivariable model. By contrast, IP (*P* = 0.011), clinical stage (*P* = 0.19), and BED10 (*P* = 0.11) met the entry threshold (*P* < 0.20) and were included. In the final multivariable model, only IP remained a statistically significant independent prognostic factor for OS (HR: 3.54, 95% CI: 1.33–9.41, *P* = 0.011) (Fig. 3).

**Table 2:** Analysis of clinical and dosimetric variables associated with OS

Variable		Univariable		Multivariable	
		*P* value	HR (95% CI)	*P* value	HR (95% CI)
Age	(> 80 vs ≤ 80)	0.52	1.28 (0.60–2.75)		
Sex	(male vs female)	0.39	0.65 (0.25–1.73)		
PS	(1–3 vs 0)	0.97	1.01 (0.47–2.20)		
IP	(yes vs no)	0.011*	3.54 (1.33–9.41)	0.011*	3.54 (1.33–9.41)
Location	(central vs peripheral)	0.67	1.21 (0.51–2.87)		
Diagnosis	(histological vs clinical)	0.58	1.75 (0.24–13.0)		
Clinical stage	(III vs II)	0.19	1.69 (0.77–3.69)	0.13	1.84 (0.84–4.04)
Dose prescription	(BED10 ≤ 100 Gy vs > 100 Gy)	0.11	0.48 (0.19–1.19)	0.27	0.59 (0.23–1.50)

HR, hazard ratio; PS, performance status; IP, interstitial pneumonia; BED, biologically effective dose; BED10, biologically effective dose with α/β = 10 Gy. *P* values are two-sided. *P < 0.05. Variables with *P* < 0.20 in univariable analysis were entered into the multivariable model; statistical significance was defined as two-sided *P* < 0.05

### Adverse events

Among the 55 patients, grade 2 and grade 3 radiation pneumonitis occurred in three (5.5%) and two (3.6%) patients, respectively, with no cases of grade ≥ 4 events. Notably, none of the nine patients with comorbid IP developed grade 2 or 3 radiation pneumonitis. No other late adverse events of grade 3 or above were reported.

## Discussion

### CIRT monotherapy for CCRT- ineligible LA-NSCLC

CCRT followed by consolidation with ICIs is the current gold standard for LA-NSCLC. In the PACIFIC trial, the 2-year OS rate improved from 55.6% in the CCRT arm to 66.3% in the CCRT + durvalumab arm [1]. However, patients enrolled in such trials are highly selected, and many individuals in real-world clinical settings are ineligible for CCRT due to factors such as age, complications, or disease status. For these patients, radiotherapy alone remains the only feasible option, though its outcomes are often suboptimal. Historical photon radiotherapy trials conducted in the 1990s and 2000s reported 2-year OS rates of 10–20% with radiotherapy alone [14, 15]. A Japanese clinical trial (JCOG0301) in elderly patients with advanced age and LA-NSCLC reported a 2-year OS of 35.1% for radiotherapy alone, compared with 43% for CCRT, using conventional radiotherapy techniques available at the time [16].

Particle beam therapy represents a promising alternative to conventional radiotherapy, potentially addressing these unmet clinical needs. While proton beam therapy for LA-NSCLC is increasingly used in Western countries, often in combination with systemic therapy, CIRT has been developed primarily in Japan and is administered as monotherapy. Most previous studies have been retrospective reports from a single or small number of institutions in Japan. The largest of these involved 141 patients treated between 1995 and 2015, reporting 2-year OS and LC rates of 58.7% and 80.3%, respectively; grade 3 pneumonitis, in 3.5% of patients; and one grade 4 adverse event (mediastinal hemorrhage) in 0.7% [17]. In another retrospective study from three institutions, the 2-year OS and LC rates were 62.2% and 81.8%, respectively, with no grade ≥ 2 adverse events observed [18].

To our knowledge, this is the first nationwide, prospective registry study evaluating CIRT alone for LA-NSCLC. In the current study, the 2-year OS and PFS rates were 66.0% and 37.5%, respectively. CIF-based local failures at 2 and 3 years were 34.8% and 37.4%, respectively. These outcomes are broadly consistent with prior Japanese CIRT monotherapy series using similar protocols and reflect standardized practice nationwide; they appear lower than some single-institution retrospective reports, which likely reflects broader eligibility (including CCRT-ineligible patients and comorbid IP), the multicenter nature of the study, and methodological differences (competing-risks vs Kaplan–Meier).

### The potential of CIRT in combination with systemic therapy

When combined with systemic therapy, CIRT may further improve outcomes in eligible patients, although toxicity must be carefully considered. A recent study of CIRT (median 77 Gy/22 fractions) combined with various systemic therapies in patients with stage III NSCLC reported favorable 2-year OS and LC of 64.2% and 66.1%, with low rates of grade ≥ 3 tracheobronchial and esophageal events (1.7% and 2.8%, respectively) [19]. In general, the risk of radiation-induced complications in the esophagus and trachea increases when chemotherapy is used and should be carefully considered even with sequential administration.

Although randomized trials such as PACIFIC-2 did not show a survival benefit for concurrent ICI with CCRT, accumulating data support the safety of combining ICIs with radiotherapy [20, 21]. For patients with LA-NSCLC who are unable to undergo chemoradiotherapy, similar to the patients in this study, these observations support immunotherapy–radiotherapy as a promising option. Moreover, in photon radiotherapy, improved survival has been reported when ICIs are given after stereotactic body radiotherapy (SBRT) in patients with early-stage lung cancer. While comprehensive clinical data for CIRT are limited, a stronger synergistic effect with ICIs is theoretically plausible and warrants prospective evaluation [22, 23]. Consequently, even frail patients who are ineligible for conventional chemotherapy, may experience extended survival, as observed in this study. Further clinical trials building on these findings for CIRT monotherapy are warranted.

### Adequacy and safety of prescribed doses

The main prescription dose used in this study was 64–72 Gy in 16 fractions under Japan’s unified treatment policy—a prescription with a higher biological effect than conventional photon regimens. Despite this, the toxicity profile was also acceptable (no grade ≥ 4 events; grade 3 pneumonitis in 3.6%), supporting the feasibility of CIRT monotherapy in a CCRT-ineligible population. The relatively low incidence of pneumonitis is consistent with the characteristic dose distribution of carbon ions [24, 25]; however, delivered OAR doses were not analyzed, so no causal inference is made. In photon radiotherapy for LA-NSCLC, various hypofractionated regimens—with or without chemotherapy—have been explored [26–28]; one randomized clinical trial comparing 60 Gy in 15 vs. 30 fractions did not show a benefit with hypofractionation [24]. While some studies report improved survival with hypofractionation, increased toxicity has also been observed, underscoring the need for careful management of esophagitis and pneumonitis [25, 26]. Future protocol-level analyses integrating delivered tumor and OAR dosimetry are warranted to clarify dose–response and dose–toxicity relationships—work that will inform not only CIRT but radiotherapy practice more broadly.

The present study included nine cases (16%) with IP, none of whom developed symptomatic radiation pneumonitis. Prior reports in early-stage NSCLC suggest a relatively low incidence of pneumonitis with CIRT in patients with concomitant IP [29, 30], and CIRT may similarly expand eligibility for curative treatment in NSCLC. At the same time, IP was the only OS-related factor identified; in patients with IP, prognosis may be more strongly influenced by the underlying lung disease than by cancer progression itself [30, 31]. Even in the absence of severe acute or subacute pneumonitis, radiotherapy may still adversely influence prognosis in patients with IP; therefore, careful risk assessment and patient selection are essential, and ongoing data collection on risk factors and indications is needed.

### Limitations

This study has several limitations. First, the sample size was modest and the observation period was short, which may have limited statistical power. As a prospective observational registry without strict standardization of treatment indications, some selection bias is possible, and the limited number of CIRT centers and the associated costs may restrict generalizability. Second, although conducted under a common treatment policy, the details of dose prescription and treatment planning varied across centers, and multiple regimens were used. Dose–volume histogram (DVH) data for lungs, heart, and esophagus were not collected, precluding analysis of dose–toxicity relationships and dose constraints; accordingly, although tumor location was examined as a prognosticator, its clinical relevance—particularly any association with lung dose—could not be assessed. Third, detailed data on adverse events other than pneumonitis and comprehensive patient background factors were not collected, limiting risk-factor analyses; the cause of death was also unavailable, which affects interpretation, particularly in patients with IP. Finally, there is no established method for directly comparing the biological effects of CIRT with photon radiotherapy, complicating cross-modality comparisons. Further multicenter studies and protocol-level analyses are needed to clarify the optimal role and treatment strategies of CIRT in LA-NSCLC.

## Conclusion

We report the nationwide prospective registry results of CIRT for LA-NSCLC in Japan. Among patients ineligible for surgery or CCRT, CIRT monotherapy showed encouraging survival with an acceptable safety profile, supporting its feasibility as a treatment option for patients with frailty or significant comorbidities. While the favorable local control support the therapeutic value of CIRT, the limitations of monotherapy suggest that outcomes may be further improved through protocol-level refinement and the strategic integration of systemic therapies. These data provide a solid basis for such efforts.

## Supplementary Information

Below is the link to the electronic supplementary material.

Supplementary Material 1
