# Supplementary figures and images for: Carbon-Ion radiotherapy alone for inoperable locally advanced Non-Small cell lung cancer: A Japanese National registry study (J-CROS-LUNG)
Source: Jpn J Radiol. 2026 Jan 13;44(4):730–7. doi: 10.1007/s11604-025-01925-z (PMC13038780; doi:10.1007/s11604-025-01925-z)

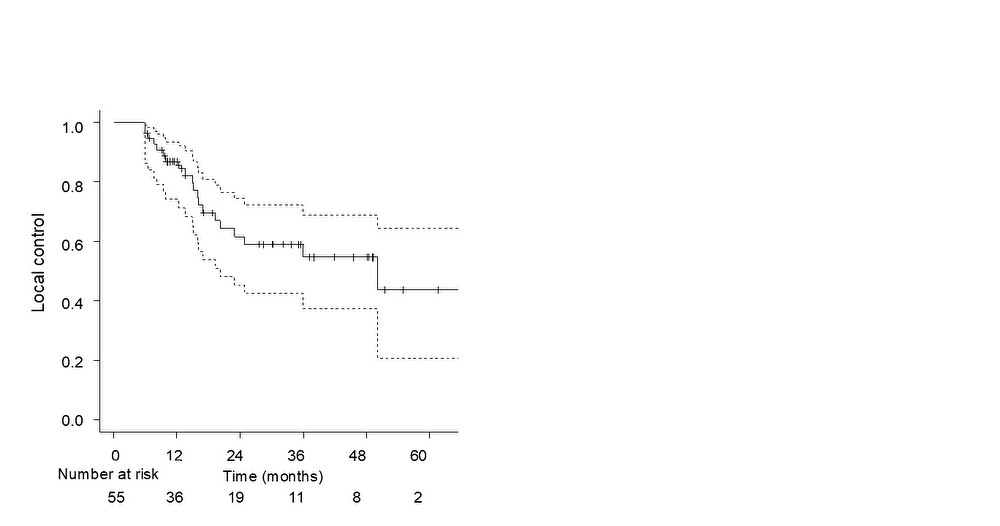

Supplement: Supplementary file 1 — Supplementary Material 1 [file 11604_2025_1925_MOESM1_ESM.jpg]
